# CDH11 Contributes to Bladder Cancer Progression via Regulation of Mitochondrial Energy Metabolism

**DOI:** 10.1002/cam4.71399

**Published:** 2025-11-20

**Authors:** Osuke Arai, Yuta Yanagihara, Haruna Arai, Ryuta Watanabe, Noriyoshi Miura, Tadahiko Kikugawa, Takashi Saika, Yuuki Imai

**Affiliations:** ^1^ Department of Urology Ehime University Graduate School of Medicine Toon Japan; ^2^ Division of Integrative Pathophysiology Proteo‐Science Center, Ehime University Toon Japan; ^3^ Department of Pathophysiology Ehime University Graduate School of Medicine Toon Japan

**Keywords:** big data analysis, bladder cancer, cancer progression, CDH11, mitochondrial energy metabolism

## Abstract

**Background:**

Approximately 83,000 new cases of bladder cancer (BC) in the United States and 23,000 in Japan are confirmed per year, and the number of new BC cases increases every year. While the prognosis for localized cancer is favorable, treatment options for metastatic cancer are limited, and the prognosis is extremely poor.

**Aim:**

Although the prevention of metastasis and the development of novel treatments for metastatic cancer are urgent challenges, the molecules contributing to BC metastasis and prognosis remain largely unknown. This study aimed to identify and analyze novel therapeutic target molecules for metastatic BC.

**Materials and Methods:**

We collected gene expression datasets for BC cell lines from Gene Expression Omnibus and selected genes highly expressed in advanced BC cell lines. We also extracted genes highly expressed in metastatic BC patients from The Cancer Genome Atlas. We performed integrated analysis on these genes and combined it with Kaplan‐Meier analysis to identify genes involved in BC progression. The results revealed that CDH11 is involved in bladder cancer progression. We established CDH11 knockdown (KD) using shRNA in advanced bladder cancer cell lines and performed analyses of cell proliferation, invasion, and migration; gene expression analysis via RNA‐seq; tumor formation via xenografts; metabolic analysis using the Flux analyzer; and therapeutic effect analysis using low‐molecular‐weight compounds.

**Results:**

While CDH11 KD did not alter cell proliferation, invasion, or migration in vitro, CDH11 KD significantly suppressed tumor growth in an in vivo subcutaneous xenograft mouse model. RNA‐seq revealed that CDH11 KD cells decreased the expression of genes related to mitochondrial metabolism, and a metabolic flux analyzer confirmed decreased mitochondrial activity in the KD cells. Furthermore, use of a CDH11 inhibitor resulted in decreased mitochondrial activity in vitro, and CDH11 inhibitor resulted in inhibition of tumor growth in vivo.

**Discussion:**

Our findings suggest that CDH11 is highly expressed in advanced BC and may regulate the TME by interacting with other cell types and regulating mitochondrial energy metabolism.

**Conclusion:**

These results suggest that CDH11 is involved in BC progression and is a potential therapeutic target.

## Graphical abstract

CDH11 inhibition can suppress mitochondrial respiration in advanced bladder cancer cells and xenograft tumor progression.

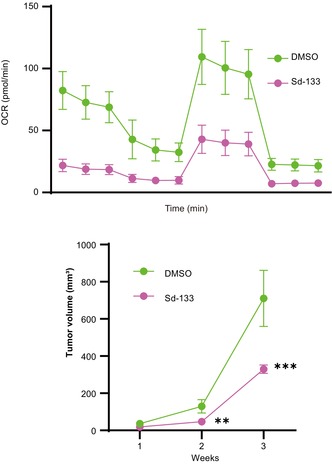

**Abbreviation**
RNA‐seq: RNA sequencing

## 1 Introduction

Approximately 83,000 new cases of bladder cancer (BC) in the United States [1] and 23,000 in Japan are confirmed per year (Cancer Statistics. Cancer Information Service, National Cancer Center, Japan [National Cancer Registry, Ministry of Health, Labour and Welfare]), and the number of new BC cases increases every year. BC is broadly classified into noninvasive and invasive carcinomas, which are genetically distinct [2]. Among the invasive carcinomas, those that invade the muscle intrinsic layer tend to progress and have a very poor prognosis. The 5‐year survival rates of BC patients are extremely low compared with other urologic cancers: 87.3% for localized cancer (nonadvanced BC), 38% for regional metastases, and 9.5% for distant metastases (both are advanced BC) [3, 4]. Localized bladder cancer is curable by transurethral resection of bladder tumor. However, if the tumor has invaded the muscle layer, the recurrence rate is high and distant metastasis is likely to occur even after total cystectomy. Thus, early detection and diagnosis of BC is important for a good prognosis. Although transcriptome analyses of BC have been conducted [5, 6], there are still no effective diagnostic markers for BC, and the difficulty of screening may lead to a delay in diagnosis. Another reason for the poor prognosis of advanced BC is that only three main treatment options are available: platinum‐based chemotherapy, immune checkpoint inhibitors, and antinectin‐4 antibody–microtubule inhibitor enfortumab vedotin. However, advanced BC is difficult to treat with chemotherapy and immune checkpoint inhibitors, and the 2‐year survival rate is 15%–25% [7]. As described above, patients with advanced BC have an extremely poor prognosis due to limited treatment options, and the development of new therapies is desired. Therefore, we aimed to identify novel therapeutic target molecules and to clarify the molecular mechanisms for treatment of advanced BC.

We hypothesized that the specific gene expression differences between nonmetastatic and metastatic BC may be involved in the transformation to advanced disease in BC. To narrow down the target genes, we used a method of omics big data analysis already validated in our laboratory [8, 9]. Among the genes identified, we focused on cadherin 11 (*CDH11*) as a gene contributing to BC progression because *CDH11* is highly expressed in advanced BC and is related to prognosis. CDH11 is a member of the cadherin family, a transmembrane protein involved in cell adhesion, and it suppresses cancer activity via the Wnt/β‐catenin, AKT/Rho A, and NF‐κB signaling pathways [10, 11, 12, 13]. There are several reports that CDH11 promotes metastasis and progression of breast and pancreatic cancers [14, 15, 16, 17], and CDH11 has been reported to be highly expressed in muscle‐invasive BC [18]. However, the effect of CDH11 on BC progression and the detailed molecular mechanisms remain to be elucidated. In this study, we demonstrate that CDH11 is involved in BC progression and that its inhibition has a tumor‐suppressive effect, suggesting that CDH11 is a potential therapeutic target molecule.

## 2 Materials and Methods

### 2.1 Integrative Analysis of Omics Big Data

Raw high‐throughput RNA‐seq data for localized BC cell lines (HT1197 and HT1376) and advanced BC cell lines (5637 and UM‐UC‐3) were obtained from the Gene Expression Omnibus (GEO) (GEO accession numbers GSE97768, GSE106637, GSE151505, and GSE186611). The localized and advanced BC cell lines were compared, and genes with higher expression in the advanced BC cells based on RaNAseq were extracted [19]. Additionally, we extracted genes from The Cancer Genome Atlas (TCGA) that were more highly expressed in primary tumors with lymph node metastasis or distant metastasis compared with localized BC. The overlapping genes among these three groups were subjected to GO enrichment analysis using Database for Annotation, Visualization and Integrated Discovery (DAVID) [20, 21].

### 2.2 Kaplan–Meier Analysis

Kaplan–Meier analysis was conducted using Kaplan–Meier Plotter software [22, 23].

### 2.3 Cell Culture

Human BC cell lines (HT1197, HT1376, 5637, and UM‐UC‐3) were purchased from the ATCC (Manassas, VA, USA). HT1197, HT1376, and UM‐UC‐3 cell lines were cultured in Minimum Essential Media (MEM; Thermo Fisher Scientific, Waltham, MA, USA) supplemented with 10% heat‐inactivated fetal bovine serum (FBS; Thermo Fisher Scientific), 1% nonessential amino acids (Thermo Fisher Scientific), 1% sodium pyruvate (Thermo Fisher Scientific), and 1% antibiotic–antimycotic (Thermo Fisher Scientific). The 5637 cell line was cultured in Roswell Park Memorial Institute 1640 medium (Fujifilm Wako, Osaka, Japan) supplemented with 10% heat‐inactivated FBS and 1% antibiotic–antimycotic. These cell lines were incubated at 37°C in a humidified atmosphere containing 5% CO_2_.

### 2.4 Real Time RT‐PCR

Total RNA was extracted using Isogen (Nippon Gene, Tokyo, Japan) and RNeasy spin column kits (Qiagen, Venlo, Nederland) and treated with DNase I (Qiagen). Subsequently, cDNA was synthesized from total RNA using PrimeScript RT Master Mix (Takara Bio Inc., Shiga, Japan). Real‐time RT‐PCR was performed using TB Green Premix Ex Taq II (Takara Bio Inc) and the Thermal Cycler Dice (Takara Bio Inc) according to the manufacturer's instructions. Gene expression levels were normalized to those of the housekeeping gene *RPLP0*. Primer sequences for each gene are listed in the Additional Files (Table S1).

### 2.5 Establishment of *CDH11* Knockdown (KD) Cell Lines by shRNA

UM‐UC‐3 and 5637 cells (1 × 10^4^) were seeded in 24‐well plates. Cells were infected with lentiviral particles containing both shRNA and GFP (Nonsilencing‐GIPZ lentiviral shRNA control [V22093005; shCtrl], Human GIPZ lentiviral shRNA individual clone [target sequence: GCCAAGTTAGTGTACAGTA; V2LHS_150470; sh#1], and/or Human GIPZ lentiviral shRNA individual clone [target sequence: TGACATGTAAGAAAATGTT; V3LHS_400950; sh#2]; Horizon Discovery Ltd., Cambridge, UK) at a multiplicity of infection of 10 for 48 h in the presence of polybrene (2 μg/mL). To collect the infected cells, GFP‐positive cells were sorted by FACS (FACSAria II, BD Biosciences, Oak Park, MA, USA). The collected cells were used for subsequent experiments.

### 2.6 CDH11 Inhibitor

4‐(2‐phenylpyridin‐3‐yl) phenol (Sd‐133, PBMR363044; Princeton BioMolecular Research Inc., Princeton, NJ, USA) binds specifically to the CDH11 binding pocket to inhibit CDH11. Sd‐133 was dissolved in DMSO (1 M, Fujifilm Wako) and stored at −20°C. Sd‐133 was used at a final concentration of 100 μM in vitro [24] and administered at 40 mg/kg (2 mg/mL in saline containing 20% Solutol HS‐15 [MedChemExpress, Monmouth Junction, NJ]) in vivo [25].

### 2.7 Cell Counting Assay

Ctrl and *CDH11* KD cell lines were seeded in six‐well plates at 1 × 10^4^/well. The number of cells was counted 72 h after seeding using a Countess cell counter (Thermo Fisher Scientific).

### 2.8 Invasion Assay

Invasion ability was evaluated using the Corning BioCoat Matrigel Invasion Chamber (Corning, Corning, NY, USA). shCtrl and *CDH11* KD cell lines were precultured under starvation conditions without FBS for 24 h. Subsequently, 5 × 10^4^ cells suspended in serum‐free MEM were seeded in the upper chambers, and MEM containing 10% FBS was added to the lower chambers. The cells in the chamber were fixed at 24 h after seeding, and the cells remaining on the upper membrane were removed using a cotton swab. The cells that had migrated to the lower membrane were then stained with Diff‐quick (Sysmex, Hyogo, Japan) according to the manufacturer's instructions. The cell number normalized to the membrane area was calculated from the microscopy images using the Fiji platform [26].

### 2.9 Migration Assay

Ctrl and *CDH11* KD cells were seeded in six‐well plates at 3 × 10^6^/well. Cell growth was suppressed by exposure to starvation conditions for 24 h in serum‐free MEM. Cells in each well were scratched using a 200 μL pipette tip. Thereafter, the cells were cultured for 24 h, and the number of migrating cells per equivalent unit area was measured using the Fiji platform [26].

### 2.10 Western Blotting

Ctrl and *CDH11* KD cell lines were lysed on ice using RIPA buffer containing a cocktail of protease and phosphatase inhibitors (Nacalai Tesque Inc., Kyoto, Japan). Samples were then applied to 10% SuperSep Ace (Fujifilm Wako) and subjected to SDS–polyacrylamide gel electrophoresis. Samples were transferred to PVDF membranes (Bio‐Rad Laboratories, Berkeley, CA, USA) using a mini transblot cell (Bio‐Rad Laboratories). The membranes were then blocked. The membranes were reacted with primary antibodies (anti‐CDH11 [Thermo Fisher Scientific; 71–7600; 1:500 dilution] and anti‐β‐actin [MBL, Tokyo, Japan; M177‐3; 1:2000 dilution]) at 4°C overnight. After washing, membranes were incubated with secondary antibodies (horseradish peroxidase [HRP]‐conjugated antirabbit IgG [Dako Agilent, Santa Clara, CA, USA; 1:2500 dilution] and HRP‐conjugated antimouse IgG [Promega, Madison, WI, USA; 1:10000 dilution]). Luminescence signal was detected using the Amersham ImageQuant800.

### 2.11 CDH11 Overexpression (O/E)

Sh#1 *CDH11* KD cells were cultured for 2 days following transfection with a CDH11 overexpression plasmid using Lipofectamine 3000 (Thermo Fisher Scientific).

### 2.12 Cell Metabolism Assay

Metabolic flux was measured using the Seahorse XFp Flux Analyzer (Seahorse Bioscience, North Billerica, MA). shCtrl, *CDH11* KD cell lines, sh#1 *CDH11* KD cell line + mock and sh#1 *CDH11* KD cell line + *CDH11* O/E were seeded into an eight‐well Seahorse culture plate (5 × 10^4^ cells/well) in MEM and precultured for 24 h. For analysis of the oxygen consumption rate (OCR), cells were cultured for 1 h in XF‐DMEM (Agilent, Santa Clara, CA, USA) supplemented with 10 mM glucose, 1 mM pyruvate and 2 mM glutamine (Seahorse Bioscience, pH = 7.4 ± 0.1) and equilibrated at 37°C in a CO_2_‐free atmosphere. After three basal measurements, 1 μM oligomycin, 1 μM carbonyl cyanide 4‐(trifluoromethoxy) phenylhydrazone (FCCP), and 0.5 μM antimycin A/rotenone were sequentially injected into the plate. For analysis of the extracellular acidification rate (ECAR), cells were cultured for 1 h in XF‐DMEM supplemented with 2 mM glutamine and equilibrated at 37°C in a CO_2_‐free atmosphere. After three basal measurements, 10 mM glucose, 1 μM oligomycin, and 25 mM 2‐DG were sequentially injected into the plate. For experiments using the CDH11 inhibitor, cells were precultured in 10 cm dishes for 60 h and treated with DMSO or 100 μM Sd‐133. Cells were then trypsinized, collected, and seeded in eight‐well Seahorse culture plates (5 × 10^4^ /well) in DMEM supplemented with 10 mM glucose, 1 mM pyruvate, and 2 mM glutamine for 2 h and equilibrated at 37°C. For the OCR measurements, 1 μM oligomycin, 1 μM FCCP, and 0.5 μM antimycin A/rotenone were injected sequentially into the plates.

### 2.13 Animals

BALB/cAJcl‐nu/nu male mice (nude mice) were purchased from CLEA Japan (Kanagawa, Japan). Mice were housed in a specific pathogen‐free facility under climate‐controlled conditions and a 12‐h light/dark cycle and were provided water and a standard diet (MF; Oriental Yeast, Tokyo, Japan) *ad libitum*. All animals were maintained and used according to the experimental protocol approved by the Animal Experiment Committee of Ehime University, Japan.

### 2.14 Tumor Xenografts

Ctrl, *CDH11* KD and UM‐UC‐3 cell lines were suspended in Matrigel solution (Corning; 1 × 10^7^ cells/ml). The cell suspension (100 μL) was inoculated subcutaneously into 5‐week‐old nude mice. Tumor volumes were calculated based on the tumor diameter measured using an electronic caliper every week (Tumor volume = 4/3 × 3.14 × (length/2 × width/2 × height/2)). Tumor tissues were sampled 4 4 weeks after transplantation. Administration of inhibitors, a solution of the Sd‐133 (40 mg/kg) or an equal volume of DMSO in saline was administered intraperitoneally three times a week [25]. Tumor volumes and body weights were measured every week. Tumor tissues were sampled 3 3 weeks after transplantation.

### 2.15 RNA‐Seq

shCtrl and *CDH11* KD cell were precultured in 12‐well plates (2 × 10^5^/well) for 24 h. Total RNA was extracted using RNeasy spin column kits and DNase I. The RNA integrity number was verified using the Agilent 2100 Bioanalyzer. RNA‐seq was performed on the Illumina NextSeq 500, with a read configuration of 75 bp for single reads; 3 million reads were generated per sample. FASTQ files were trimmed using CLC Genomics Workbench (Filgen, Aichi, Japan). Mapping of FASTQ files to the human genome assembly hg38 was performed using RaNAseq [19]. Genes with a Log_2_FC value > 1 or < −1 and *q* value < 0.05 were selected for GO enrichment analysis using Metascape [27]. Tumor tissue pieces sampled from the xenograft model were lysed using ISOGEN. Total RNA was extracted using RNeasy spin column kits and DNase I. RNA‐seq was performed on the NovaSeq6000, with a read configuration of 75 bp for single reads; 3 million reads were generated per sample. FASTQ files were trimmed using trim galore. Mapping of FASTQ files to the human genome assembly hg38 was performed using HISAT2 [28, 29, 30, 31]. Genes with a *q* value < 0.1 were selected for GO enrichment analysis using Metascape. Volcano plots were created using iDEP [32]. The RNA‐seq datasets were deposited in GEO under accession number GSE277038.

### 2.16 Immunocytochemical Staining

Ctrl and *CDH11* KD cell lines were fixed using 4% paraformaldehyde for 5 min, permeabilized for 10 min, and blocked using 1% bovine serum albumin in 0.02% Triton X‐PBS (blocking buffer). Cells were incubated overnight at 4°C with an anti‐Ki67 primary antibody (NOVUS, Centennial, CO, USA, NB500‐170, 1:250 dilution) diluted in blocking buffer. After washing, the cells were incubated with the AlexaFluor 568 mouse IgG secondary antibody (1:500 dilution) diluted in blocking buffer. After washing, the cells were encapsulated with DAPI‐containing encapsulant (Vector, Newark, CA, USA) and observed under a fluorescence microscope (ZEISS Microscopy ZEN3.x Pro). Ki67‐positive cell rate was calculated using the Fiji platform [26].

### 2.17 Histological Analysis

Tumor tissues obtained from the xenograft mouse models were fixed using 4% paraformaldehyde for 24 h and then embedded in paraffin. Tissue sections 3–5 μm thick were prepared using a microtome (YAMATO, Saitama, Japan). For hematoxylin and eosin (HE) staining, deparaffinized sections were stained with Carazzi's hematoxylin for 10 min, followed by staining with eosin Y for 10 min. For immunostaining, deparaffinized sections were treated with 30% H_2_O_2_ in 50% ethanol for 30 min to block endogenous peroxidase, followed by rinsing in PBS for 5 min. Then, the sections were subjected to microwave treatment, followed by PBS washes, and blocking with goat serum for 30 min at room temperature (VEC S‐1000; Vector). The primary antibody (CD31 (Cell Signaling Technology, Danvers, MA), 1:400 dilution) reaction was performed overnight at 4°C, followed by PBS washes. The sections were treated with Histofine Simple Stain Mouse MAX‐PO(R) (Nichirei Biosciences Inc., Tokyo, Japan) to conjugate HRP with the primary antibody and then washed with PBS. The peroxidase signal was detected using 3,3′‐diaminobenzidine, and the nuclei were stained with Mayer's hematoxylin (Sakura Finetek Japan, Tokyo, Japan). Stained tissue sections were photographed using the BZ‐X800, and image analysis was performed using the Fiji platform [26].

### 2.18 Morphological Observation of Mitochondria

Mitochondrial morphology was observed using a confocal laser microscope (Nikon, Tokyo, Japan; A1Rsi‐E1). The nucleus was stained with Hoechst (Thermo Fisher Scientific), and the mitochondria were stained with MitoBright LT Red (Dojindo Molecular Technologies Inc. Kumamoto, Japan).

### 2.19 Statistical Analysis

The unpaired *t*‐test for comparisons between two groups and ordinary one‐way ANOVA followed by Dunnett's test for comparisons among three groups was performed using GraphPad Prism 10 (GraphPad Software). In all graphs, data are represented as means ± standard deviations. Statistical significance was indicated by a *p* value < 0.05.

## 3 Results

### 3.1 *CDH11* Is Highly Expressed in Advanced BC

**FIGURE 1:**
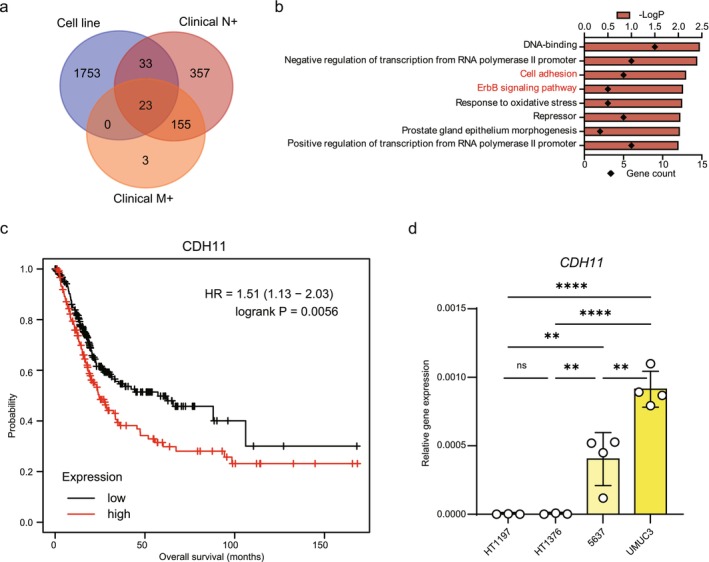
Differences in gene expression between localized and advanced BC. (a) Venn diagram showing the genes with higher expression in advanced BC using GEO and TCGA data. Cell line: Higher expression in advanced BC cell lines, Clinical N+: Higher expression in primary tumors with lymph node metastasis, Clinical M+: Higher expression in primary tumors with distant metastasis. (b) Results of GO enrichment analysis of 23 overlapping genes. (c) Kaplan–Meier curve showing a correlation between high *CDH11* expression and worse overall survival in BC patients. (d) *CDH11* mRNA levels in HT1197, HT1376, 5637, and UM‐UC‐3 cells (*n* = 3). Data represent means ± SD. ** and **** indicate *p* < 0.01 and *p* < 0.0001, respectively (one‐way ANOVA followed by Dunnet's test).

Localized and advanced BC are recognized as genetically distinct cancers [2]. Therefore, we hypothesized that genes specifically expressed in advanced BC can serve as therapeutic targets. We performed an integrative analysis of omics big data obtained from GEO and TCGA. Using this method, we identified GPRC5A as a molecule involved in the bone metastasis of prostate cancer and found that LIM1 contributes to the malignant potential of endometrial cancer [8, 9]. Using RNA‐seq data for HT1197 and HT1376 cells, as localized BC cell lines, and 5637 and UM‐UC‐3 cells, as advanced BC cell lines, we extracted 1809 genes that are significantly upregulated (with Log_2_FC ≥ 1 and *q* value < 0.01) in the advanced BC cell lines (Figure 1a, blue circle). Furthermore, according to RNA expression data for BC patients from TCGA, which contains cancer‐related gene expression data for many clinical patients, 568 genes highly expressed in patients with lymph node metastasis (Figure 1a, red circle) and 181 genes highly expressed in patients with distant metastasis (Figure 1a, orange circle), compared with localized cancer, were identified, respectively. Of these, 23 genes were common among these three groups (Figure 1a). GO enrichment analysis of these genes was performed using DAVID. We focused on eight genes enriched in both the “cell adhesion” and “ErbB signaling” GO terms, which are involved in cancer metastasis (Figure 1b). Next, we performed a prognostic analysis of the eight genes using the Kaplan–Meier plotter tool to clarify their clinical significance. From the results, we focused on *CDH11*, which was associated with a high Hazard Ratio (HR) and poor prognosis (Figure 1c). To confirm high expression of *CDH11* in advanced BC cell lines as well as GEO data analysis, RT‐qPCR was performed in the HT1197, HT1376, 5637, and UM‐UC‐3 cell lines. *CDH11* was highly expressed in the advanced BC cell lines (5637 and UM‐UC‐3) compared with the localized BC cell lines (HT1197 and HT1376) (Figure 1d). These findings suggest that *CDH11* may contribute to the progression of BC.

### 3.2 *CDH11* Deficiency in BC Cells Has no Phenotypic Effect In Vitro

**FIGURE 2:**
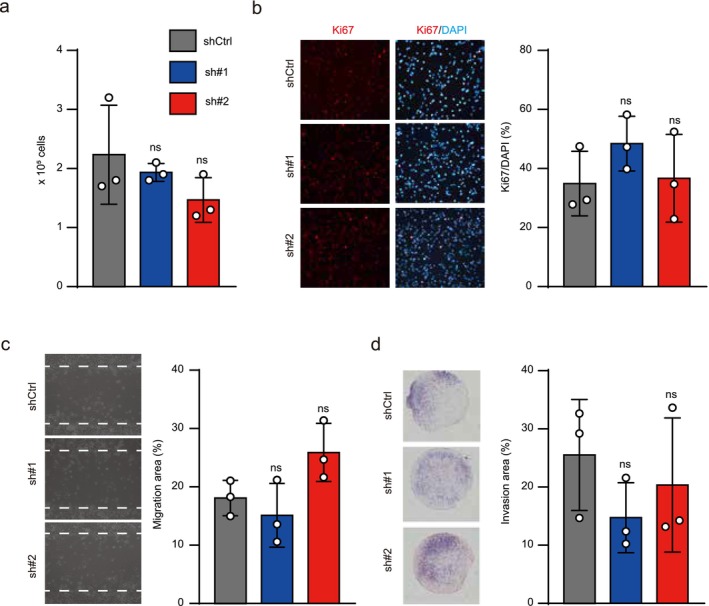
Deficient *CDH11* expression did not affect the tumor cell phenotype in vitro. (a) Cell counting assay in control (shCtrl) and *CDH11* KD (sh#1 and sh#2) cells (*n* = 3). (b) Left panel: Representative immunocytochemical analysis of Ki67 (red) and DAPI (blue) in shCtrl and *CDH11* KD cells. Right panel: Population of Ki67‐positive cells among DAPI‐positive cells in shCtrl and *CDH11* KD cell lines (*n* = 3). (c) Left panel: Cell migration activity measured by scratch assay in shCtrl and *CDH11* KD cell lines (*n* = 3). Right panel: Quantification of cell migration areas. (d) Invasion assays in shCtrl and *CDH11* KD cell lines (*n* = 3). Left panel: Representative photographs are shown. Right panel: Quantification of cell invasion areas. Scale bar: 100 μm (b, c) and 500 μm (d). Data represent means ± SD. ns: Not significant (one‐way ANOVA followed by Dunnet's test).

To investigate how CDH11 affects BC progression, we generated *CDH11* KD cell lines using different sequences of shRNAs (sh#1, and sh#2) specific for *CDH11* in the advanced BC cell line UM‐UC‐3. CDH11 mRNA and protein expression levels were significantly reduced in *CDH11* KD cells compared with shCtrl cells (Figure S1a,b). To assess cell proliferation, a cell counting assay and Ki67 immunostaining were performed. The cell numbers (Figure 2a) and proportion of Ki67‐positive cells (Figure 2b) were comparable between the shCtrl and *CDH11* KD cells. Next, cell invasion and migration assays were performed, but there was no significant difference in cell invasion or migration between the shCtrl and *CDH11* KD cell lines (Figure 2c,d). These results indicated no role of CDH11 in tumor progression in vitro, which differs from the biological environment.

### 3.3 CDH11 Suppression Inhibits Tumor Growth In Vivo

**FIGURE 3:**
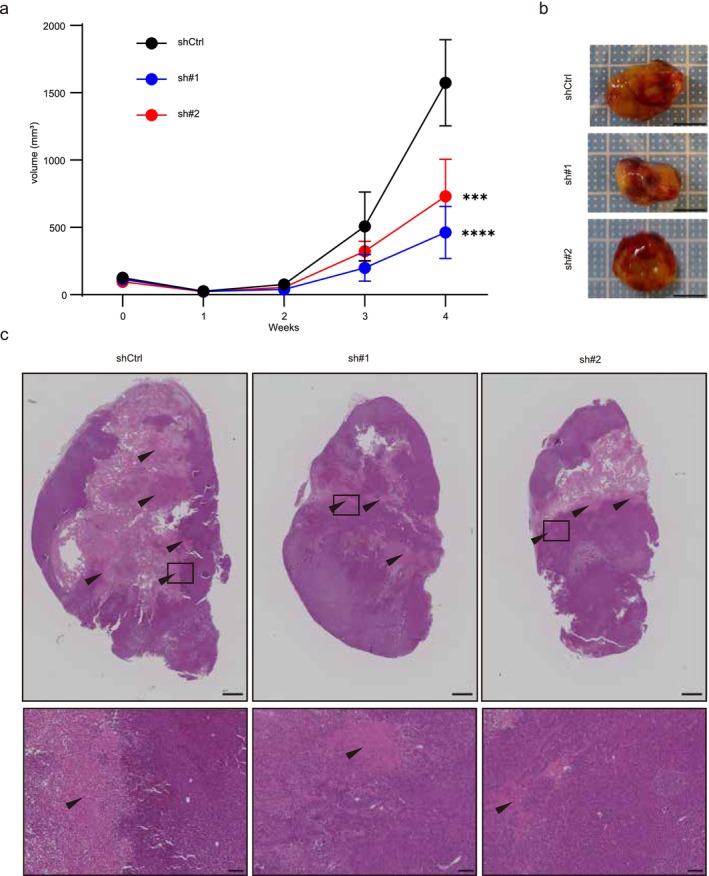
Deficient *CDH11* expression in UM‐UC‐3 suppressed tumor growth in vivo. (a) Subcutaneous size of tumors derived from control (shCtrl) and *CDH11* KD cell lines over time. (b) Excised tumors at 4 weeks after inoculation of shCtrl and *CDH11* KD cell lines. (c) HE staining of tumor tissue sections. Lower panels show a high‐magnification view of the boxed areas in the upper panels (*n* = 5). The arrow heads indicate the necrotic area. Scale bar: 1 mm (upper) and 100 μm (lower). Data represent means ± SD. *** and **** indicate *p* < 0.001 and *p* < 0.0001 compared with shCtrl, respectively (one‐way ANOVA followed by Dunnet's test).

It has also been reported that CDH11 induces collagen and elastin synthesis, and promotes extracellular matrix (ECM) synthesis [33]. CDH11 is highly expressed in cancer‐associated fibroblasts, which form part of the tumor microenvironment (TME), and inhibition of CDH11 inhibited tumor migration in vivo [34]. Based on those reports, we wanted to evaluate tumors under conditions close to the actual tumor environment in vivo despite no changes in cell proliferation, invasion, or migration observed in vitro (Figure 2). Therefore, we used a subcutaneous xenograft model to examine the effects on tumor growth in vivo, where various cell types are present. In contrast to the in vitro experimental results, percutaneously evaluated tumor size was significantly smaller at 4 weeks in xenograft tumors derived from both sh#1 and sh#2 *CDH11* KD cells compared with shCtrl cells (Figure 3a,b). HE staining of tumor sections showed broad necrotic areas in shCtrl tumors but limited areas in sh#1 and sh#2 tumors (Figure 3c). These results suggested that suppression of *CDH11* expression inhibited tumor growth in vivo.

### 3.4 *CDH11* Deficiency Suppresses Mitochondrial Metabolism

**FIGURE 4:**
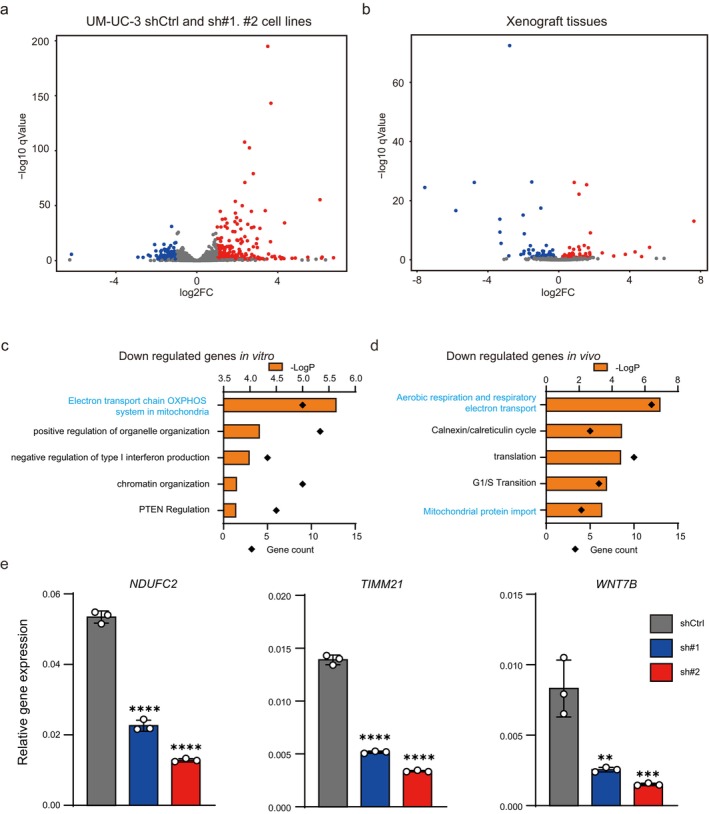
Deficient *CDH11* expression suppressed that of mitochondrial energy metabolism–related genes. (a) Volcano plot of the differentially expressed genes in *CDH11* KD (sh#1 [*n* = 3] and sh#2 [*n* = 3]) versus shCtrl (*n* = 3) based on the RNA‐seq results from cultured cells. (b) Volcano plot of the differentially expressed genes in *CDH11* KD (sh#1 [*n* = 5] and sh#2 [*n* = 5]) versus shCtrl (*n* = 5) based on the RNA‐seq results from xenograft tumor tissues. (c) GO enrichment analysis of the downregulated genes in *CDH11* KD compared with shCtrl from cultured cells. (d) GO enrichment analysis of the downregulated genes in *CDH11* KD compared with shCtrl from xenograft tumor tissues. (e) Validation of the mRNA expression of the downregulated genes in cultured cells (*n* = 3). Data represent means ± SD. **, ***, and **** indicate *p* < 0.01, *p* < 0.001, and *p* < 0.0001 compared with shCtrl cells, respectively (one‐way ANOVA followed by Dunnet's test).

**FIGURE 5:**
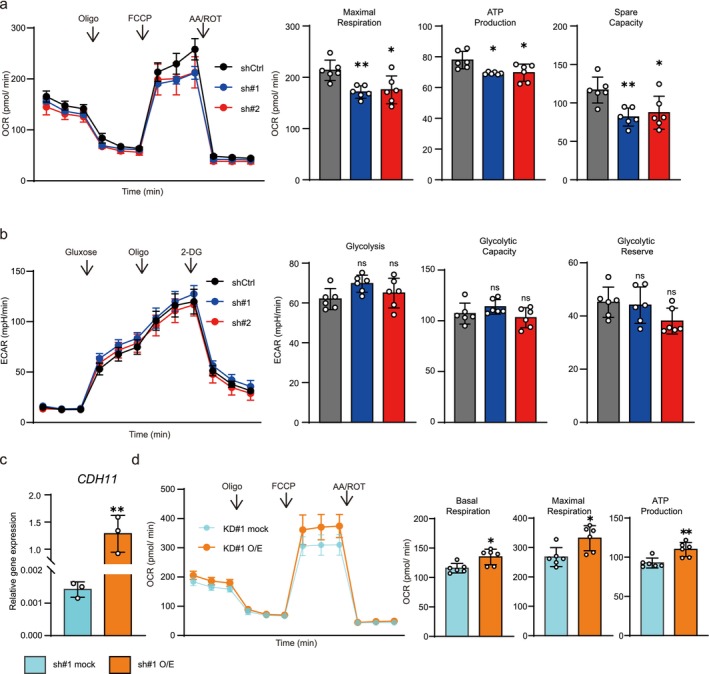
Suppression of *CDH11* reduced mitochondrial metabolism. (a) Left panel: Oxygen consumption rate (OCR) was assessed after the addition of oligomycin (Oligo), carbonyl cyanide 4‐(trifluoromethoxy) phenylhydrazone (FCCP), and rotenone/antimycin A (ROT/AA) at the indicated times. Right panel: Maximal respiration, ATP production, and spare capacity were calculated (*n* = 6). (b) Left panel: Extracellular acidification rate (ECAR) was assessed after the addition of glucose, Oligo, and 2‐DG at the indicated times. Right panel: Glycolysis, glycolytic capacity, and glycolytic reserve were calculated (*n* = 6). (c) *CDH11* mRNA levels in KD cells and CDH11 O/E in KD cells. (d) Left panel: OCR was assessed after the addition of Oligo, FCCP, and ROT/AA at the indicated times. Right panel: Basal Respiration, Maximal respiration and ATP production were calculated (*n* = 6). Data represent means ± SD. * and ** indicate *p* < 0.05 and *p* < 0.01 compared with shCtrl, respectively. ns: Not significant change (one‐way ANOVA followed by Dunnet's test in figure a and b, unpaired *t*‐test in figure c and d)

To elucidate the detailed molecular mechanisms of CDH11‐mediated BC progression, RNA‐seq was performed using shCtrl and *CDH11* KD cultured cells and xenograft tumor tissues. In cultured cells, gene expression visualized using volcano plots showed that 743 and 483 genes were significantly upregulated and downregulated, respectively, in *CDH11* KD cells. Among them, 117 and 63 genes were more than two‐fold, respectively (Figure 4a). Recently, the bladder cancer taxonomy group suggested the consensus molecular classification of BC [35, 36]. In these reports, the HT1197 (lowest *CDH11* expression) cell line was a luminal subtype cell line and the UM‐UC‐3 (highest *CDH11* expression) cell line was a basal subtype cell line. The following genes were not included in the differentially expressed genes of RNA‐seq analysis, suggesting that *CDH11* KD did not induce changes in molecular subtypes: Luminal marker (KRT20, PPARG, FOXA1, GATA3, SNX31, VPK1A, VPK2, FGFR3), Luminal infiltrated marker (PGM5, DES, C7, SFRP4, COMP, SGCD), Basal marker (CD44, KRT6A, KRT5, KRT14, COL17A1), Immune marker (CD274, PDCD1LG, IDO1, CXCL11, LICAM, SAA1), Squamous marker (DSC3, GSDMC, TGM1, PI3, TP63). In xenograft tumor tissues, 75 and 81 genes were upregulated and downregulated with *q* values less than 0.1, respectively (Figure 4b). To examine the biological pathways that CDH11 may regulate, GO enrichment analysis was performed on these gene groups in both cultured cells (Figure 4c,d) and xenograft tumor tissue results (Figure S2a,b). Mitochondria and respiration‐related genes were the most frequently enriched gene groups that were downregulated in both cultured cells and xenograft tumor tissues (Figure 4c,d). In contrast, no overlapping pathways were found in cultured cells and xenograft tumor tissues for the upregulated genes (Figure S2a,b). To validate the mRNA expression levels of the genes related to energy metabolism (*NDUFC2, TIMM21*, and *WNT7B*), we performed RT‐qPCR. There were significant differences between the shCtrl and *CDH11* KD cells (Figure 4e). Because hypoxia‐related genes were significantly upregulated in cultured cells, suggesting that angiogenesis within the tumor was promoted. Therefore, immunostaining of the vascular endothelial marker CD31 was performed in the tumor tissues obtained from the xenograft experiment. However, there was no difference in the CD31‐positive vascular endothelial area between the shCtrl and *CDH11* KD cells (Figure S2c,d). Next, energy metabolism was evaluated in the shCtrl, *CDH11* KD, and *CDH11* O/E in KD cells because metabolism‐related genes such as those involved in the OXOPHOS pathway were significantly downregulated. Since both mRNA and protein expression of CDH11 were lower in sh#1 *CDH11* KD cells, O/E was performed in sh#1 *CDH11* KD cells. First, we observed the morphology of mitochondria and cells using a confocal laser microscope, but no significant changes were observed in shCtrl + mock, sh#1 *CDH11* KD cells + mock or *CDH11* O/E (Figure S3). Then, an extracellular flux analyzer revealed that mitochondrial energy metabolism was significantly downregulated in both sh#1 and sh#2 *CDH11* KD cells compared with the shCtrl cells (Figure 5a), but there was no change in glycolysis, as indicated by the ECAR (Figure 5b). It was also confirmed that *CDH11* O/E in sh#1 *CDH11* KD cells activates mitochondrial metabolism (Figure 5c,d). To confirm whether downregulation of mitochondrial energy metabolism by *CDH11* deficiency is dependent on cell line or not, *CDH11* KD was also performed on 5637, another advanced BC cell line (Figure S4a). Mitochondrial activity was also suppressed in 5637 *CDH11* KD cells (Figure S4b,c). These results suggest that *CDH11* deficiency suppresses mitochondrial activity, resulting in reduced tumor growth in vivo.

### 3.5 A CDH11 Inhibitor Suppresses BC Tumor Growth and Mitochondrial Metabolism

**FIGURE 6:**
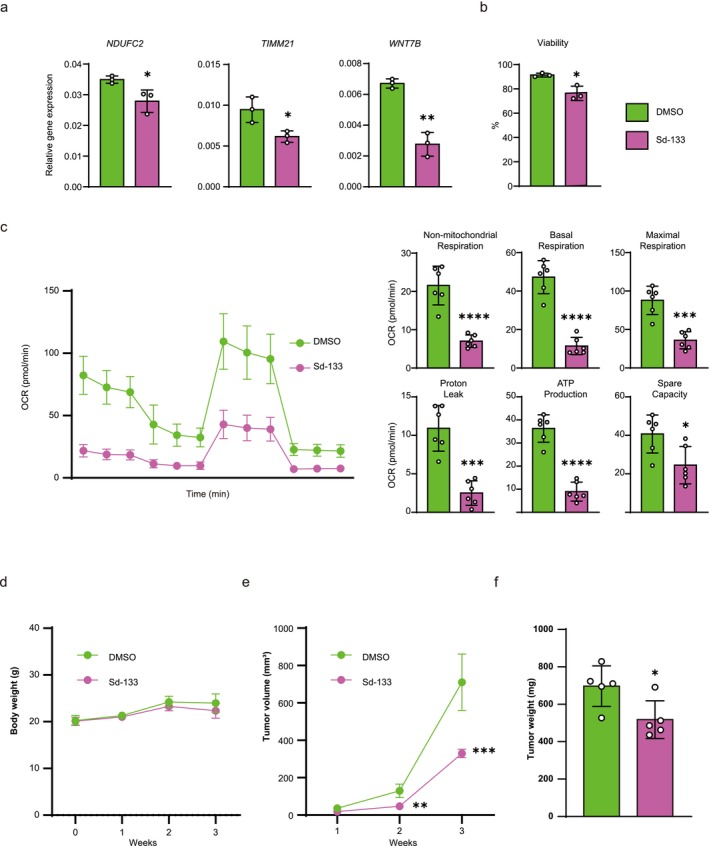
CDH11 inhibitor, Sd‐133, suppressed mitochondrial activity in vitro and tumor growth in vivo. (a) mRNA expression of the genes downregulated in *CDH11* KD cell lines (*n* = 3). (b) Cell viability under DMSO and Sd133 treatment. (c) Left panel: OCR was assessed after the addition of Oligo, FCCP, and ROT/AA at the indicated times. Right panel: Nonmitochondrial respiration, basal respiration, maximal respiration, proton leak, ATP production, and spare capacity were calculated (*n* = 6). (d) Body weight fluctuations after Sd‐133 administration (*n* = 5). (e) Changes in the sizes of subcutaneous tumors over time (*n* = 5). (f) Comparison of the weights of the extracted tumors (*n* = 5). Data represent means ± SD. *, **, ***, and **** indicate *p* < 0.05, *p* < 0.01, *p* < 0.001, and *p* < 0.0001, respectively (unpaired *t*‐test).

We evaluated tumor growth and mitochondrial energy metabolism using a CDH11 inhibitor (Sd‐133). Sd‐133 treatment downregulated the expression levels of *NDUFC2*, *TIMM21*, and *WNT7B* in UM‐UC‐3 cells, to similar levels as those in *CDH11* KD cells (Figure 6a). Cell viability decreased in the Sd‐133 treated condition (Figure 6b). Therefore, cells were precultured with DMSO and Sd‐133, and subsequently conducted metabolic experiments using the same number of living cells without DMSO and Sd‐133. Mitochondrial activity was significantly decreased in Sd‐133‐treated UM‐UC‐3 cells compared with DMSO‐treated cells (Figure 6c). These results strongly support that CDH11 inhibition regulates mitochondrial energy metabolism in advanced BC at the gene expression level. Therefore, we also examined the effect of a CDH11 inhibitor in vivo using a subcutaneous xenograft mouse model in UM‐UC‐3 cells. Sd‐133 treatment significantly suppressed tumor growth without causing weight loss and no visible changes occurred in treated mice (Figure 6d–f). These results suggest that CDH11 may be a possible therapeutic target molecule for advanced BC with fewer side effects.

## 4 Discussion

Therapeutic options for advanced BC are limited, and almost no candidate molecules for molecularly targeted agents have been reported to date. In this study, we found that CDH11 can contribute to BC progression based on big data analysis and demonstrated that inhibition of CDH11 altered tumor growth of advanced BC in vivo.

CDH11 belongs to the cadherin superfamily and is important for cell–cell adhesion. CDH11 promotes immunosuppression and ECM deposition in pancreatic cancer [17, 25]. In breast cancer, CDH11 is reportedly involved in many important biological processes by regulating the synthesis of ECM components and affecting tissue mechanical properties and contractile function [24]. Thus, our findings that *CDH11* KD cells showed no change in cell proliferation in vitro but were suppressed in vivo suggest that CDH11‐mediated cell–cell interactions between tumor cells and other cell types in the surrounding microenvironment are required for tumor growth in BC.

The Warburg effect is the phenomenon in which cancers ferment glucose in the presence of oxygen, suggesting that defective mitochondrial respiration may be the underlying cause of cancer [37]. In recent years, however, mitochondrial biogenesis and quality control are often upregulated in cancer, and removal of mitochondrial DNA suppresses tumorigenesis. Tumor growth requires the mitochondrial electron transfer chain, and inhibition of mitochondrial complex I has been reported to synergistically reduce S6K1 activity related to tumor growth and proliferation [38]. Mitochondrial complex I has also been reported to regulate the TME independently of energy depletion [39]. In our study, we observed decreased gene expression and activity related to mitochondrial metabolism in *CDH11* KD cells. These results suggest that tumor growth is suppressed in vivo only, by the direct effect of decreased CDH11 expression on the TME as well as by the effect of decreased mitochondrial activity on further suppression of TME formation. In other cancer types, CDH11 has been reported to activate the WNT/β‐catenin pathway [14], which regulates mitochondrial activity [40, 41]. Thus, *CDH11* KD cells may also have reduced mitochondrial activity via inactivation of the WNT/β‐catenin pathway.

As described above, CDH11 is important for tumor growth in vivo, and administration of CDH11 inhibitors suppressed tumor growth in the xenograft mouse model without weight loss or death. Previously, it was reported that CDH11 neutralizing antibodies slowed tumor growth and colony formation in breast, glioblastoma, and prostate cancer cells [24]. Furthermore, dual cadherin antibodies recognizing CDH1 (epithelial E‐cadherin) and CDH11 (mesenchymal OB‐cadherin) have been reported to target circulating tumor cells and inhibit bloodborne metastasis in breast and pancreatic cancers [16]. Inhibition of CDH11 has also been reported to inhibit tumor growth by suppressing the expression of β‐catenin and vimentin in mouse models of breast cancer [15].

Cancer Dependency Map (DepMap: https://depmap.org/portal.) is a web tool that searches for gene dependencies (necessary for cell survival or proliferation) in cancer cell lines by RNAi or CRISPR screening. According to DepMap, RNA silencing of *CDH11* has little effect on BC cell lines including UM‐UC‐3 and 5637 cell lines. The lack of effect of *CDH11* knockdown on cell proliferation in vitro in this study is a result that is supported by DepMap data. Similar to BC, breast and pancreatic cancer cell lines are also largely unaffected by CDH11 according to DepMap. In contrast, our findings and reports from other cancer cell lines [15, 16], reveal that suppression of CDH11 reduces tumor growth and metastasis in mouse models. These findings suggest that CDH11 may regulate cancer cell growth not among cancer cells but through interactions with TMEs and other cell types.

However, there have been no reports that the mechanism underlying CDH11 inhibition is dependent on mitochondrial suppression. Mitochondria play a central and multifunctional role in malignant tumor progression, and targeting mitochondria may have therapeutic potential [42]. Those reports already point to the importance of CDH11 in malignant tumor progression, CDH11 cell–cell interactions, and mitochondria as therapeutic targets, as well as the potential rapid clinical application of CDH11 [24].

Taken together, our findings suggest that CDH11 is highly expressed in advanced BC and may regulate the TME by interacting with other cell types and regulating mitochondrial energy metabolism. Mitochondrial activity plays an important role in the acquisition of resistance to chemotherapy in malignant tumors [43], as some reports suggest that decreased mitochondrial activity increases chemotherapy sensitivity [44]. We expect that inhibition of CDH11 may have clinical applications not only as a novel single therapy but also in combination with existing therapies.

## 5 Limitations

In this study, there was a discrepancy between the in vivo and in vitro results regarding cell growth ability. This discrepancy is considered to result from differences in signaling triggered by the binding of CDH11 expressed on the cell surface. Under in vitro conditions, cells do not interact unless they reach confluence; therefore CDH11 signaling is not activated. Since normally cell proliferation is inhibited under confluent conditions in vitro, it is difficult to evaluate proliferative capacity mediated by CDH11, which depends on cell–cell interaction.

In mouse experiments using Sd‐133, no severe side effects such as weight loss or changes in visible behavior were observed. However, the precise biological effects of CDH11 inhibitors, including Sd‐133, remain a significant issue for clinical application and require further investigation through blood tests and postmortem examinations.

No previous study has reported a direct association between CDH11 and mitochondria. The detailed molecular mechanisms underlying CDH11 regulation of mitochondrial function were not determined in this study. We will investigate the relationship between CDH11 and mitochondrial activity, focusing on the WNT/β‐catenin pathway and/or novel regulatory pathways in our future study.

## Author Contributions

**Osuke Arai:** investigation (lead), validation (lead), writing – original draft (equal). **Yuta Yanagihara:** investigation (equal), validation (equal), writing – review and editing (equal). **Haruna Arai:** investigation (equal), validation (equal). **Ryuta Watanabe:** investigation (equal), validation (equal). **Noriyoshi Miura:** supervision (equal). **Tadahiko Kikugawa:** supervision (equal). **Takashi Saika:** supervision (equal). **Yuuki Imai:** supervision (lead), writing – review and editing (equal).

## Ethics Statement

Animal Studies: Animal experiments were approved by the Ehime University Animal Experiment Committee based on the Ehime University Animal Experiment Regulations (Approval No.: 37A‐5‐16). In conducting the animal experiments, Ehime University will comply with the Law Concerning the Welfare and Management of Animals, the Standards for the Care and Keeping of Laboratory Animals and the Alleviation of Pain and Suffering, the Basic Guidelines for the Conduct of Animal Experiments at Research Institutions, etc., and the Guidelines for the Proper Implementation of Animal Experiments formulated by the Science Council of Japan.

## Consent

The authors have nothing to report.

## Conflicts of Interest

The authors declare no conflicts of interest.

## Supporting information


**Figure S1:** cam471399‐sup‐0001‐FigureS1.ai.

**Figure S2:** cam471399‐sup‐0002‐FigureS2.ai.

**Figure S3:** cam471399‐sup‐0003‐FigureS3.ai.

**Figure S4:** cam471399‐sup‐0004‐FigureS4.ai.

**Table S1:** Primer names, corresponding sequences and product sizes.

## Data Availability

The RNA‐seq datasets were deposited in GEO under accession number GSE277038. The data that support the findings of this study are available from the corresponding author upon reasonable request.
