# Supplementary material for: CDH11 Contributes to Bladder Cancer Progression via Regulation of Mitochondrial Energy Metabolism
Source: Cancer Med. 2025 Nov 20;14(22):e71399. doi: 10.1002/cam4.71399 (PMC12631170; doi:10.1002/cam4.71399)
Supplement: Supplementary file 5 — Table S1: Primer names, corresponding sequences and product sizes. [file CAM4-14-e71399-s001.docx]

Tabel S1. Primer names, corresponding sequences and product sizes.

| Primer | Sequence | | | | Product size (bp) |
| --- | --- | --- | --- | --- | --- |
| RPLP0 | F: | 5'- | AGCCCAGAACACTGGTCTC | -3' | 97 |
|  | R: | 5'- | ACTCAGGATTTCAATGGTGCC | -3' |  |
| CDH11 | F: | 5'- | AGAGAGCCCAGTACACGTTGA | -3' | 142 |
|  | R: | 5'- | TTGGCATGATAGGTCTCGTGC | -3' |  |
| NDUFC2 | F: | 5'- | CGGCCTGATTGATAACCTAATCC | -3' | 64 |
|  | R: | 5'- | AAGCTGGCGATGCAAACCA | -3' |  |
| TIMM21 | F: | 5'- | AGAAGCCGGAAGAGATTTTACCT | -3' | 167 |
|  | R: | 5'- | CACCGATCACCTCAGGATGTG | -3' |  |
| WNT7B | F: | 5'- | GAAGCAGGGCTACTACAACCA | -3' | 155 |
|  | R: | 5'- | CGGCCTCATTGTTATGCAGGT | -3' |  |

F: forward and, R: reverse primers
